# Supplementary material for: The interactome of intact mitochondria by cross-linking mass spectrometry provides evidence for coexisting respiratory supercomplexes
Source: Mol Cell Proteomics. 2017 Dec 8;17(2):216–32. doi: 10.1074/mcp.RA117.000470 (PMC5795388; doi:10.1074/mcp.RA117.000470)
Supplement: Supplemental Data [file supp_RA117.000470_133922_0_supp_23978_fzffwf.zip › spectra_annotation/mito_spectra_annotation/190-1-1-1-4-1.pdf]

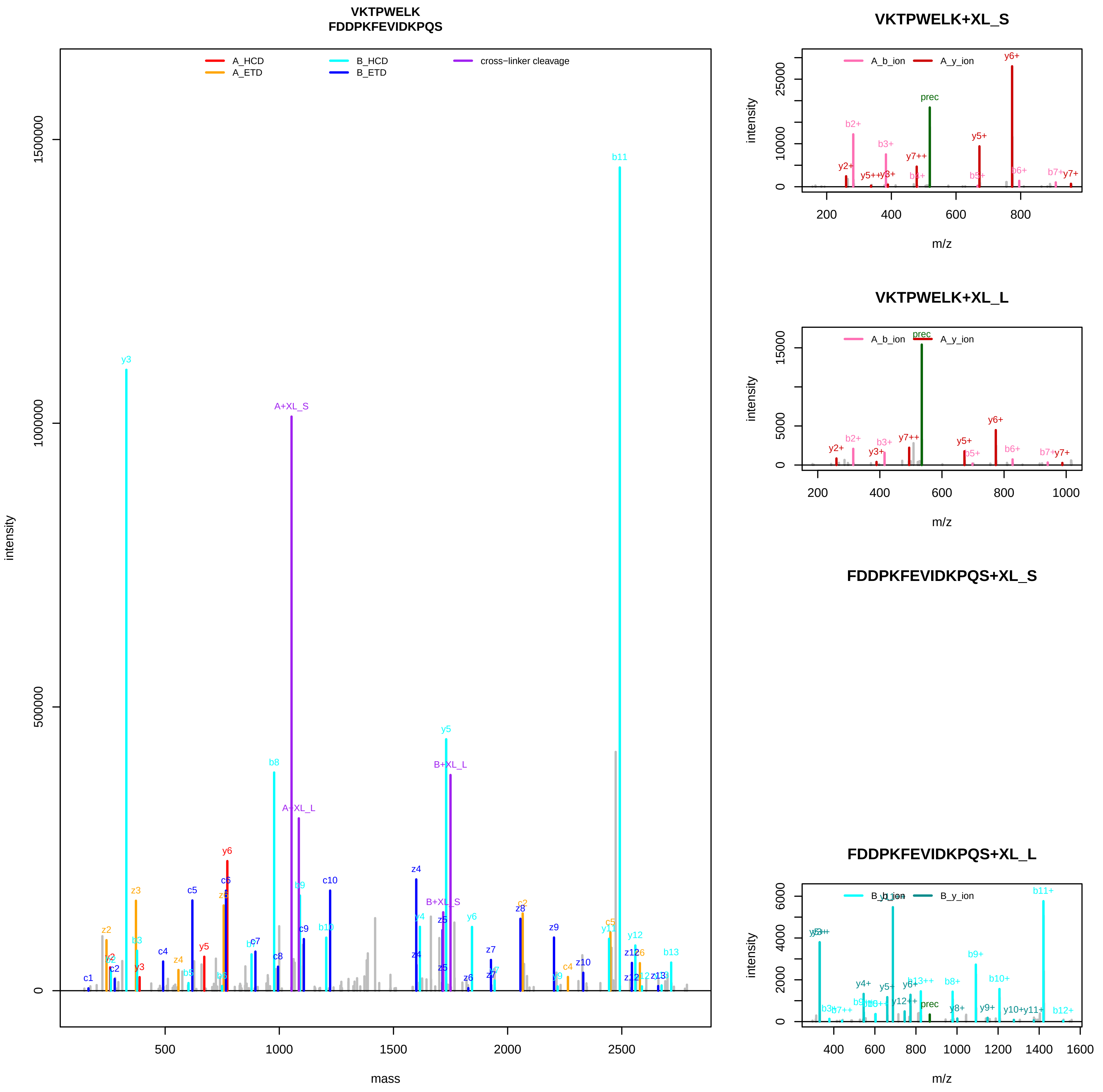

VKTPWELK
FDDPKFEVIDKPQS
A_HCD
A_ETD
B_HCD
B_ETD
cross−linker cleavage
intensity
mass
0
500000
1000000
1500000
500
1000
1500
2000
2500
b11
y3
A+XL_S
y5
B+XL_L
b8
A+XL_L
y6
c10
z4
B+XL_S
c5
c6
z3
b9
VKTPWELK+XL_S
A_b_ion
A_y_ion
prec
y6+
b2+
b3+
y5+
y7++
intensity
m/z
200
400
600
800
0
10000
25000
VKTPWELK+XL_L
A_b_ion
A_y_ion
prec
y6+
intensity
m/z
200
400
600
800
1000
0
5000
15000
FDDPKFEVIDKPQS+XL_S
FDDPKFEVIDKPQS+XL_L
B_b_ion
B_y_ion
b11+
b9+
b10+
intensity
m/z
400
600
800
1000
1200
1400
1600
0
2000
4000
6000
